# Uncovering Interoceptive Human Insular Lobe Function through Intraoperative Cortical Stimulation—A Review

**DOI:** 10.3390/brainsci14070646

**Published:** 2024-06-27

**Authors:** Pascal O. Zinn, Ahmed Habib, Hansen Deng, Neslihan Nisa Gecici, Hayat Elidrissy, Yassine Alami Idrissi, Mohammadreza Amjadzadeh, Natalie Sandel Sherry

**Affiliations:** 1 Department of Neurosurgery, University of Pittsburgh, Pittsburgh, PA 15232, USA; habiba@upmc.edu (A.H.); dengh3@upmc.edu (H.D.); n.ngecici@gmail.com (N.N.G.); sherrynk@upmc.edu (N.S.S.); 2 UPMC Hillman Cancer Center, University of Pittsburgh, Pittsburgh, PA 15232, USA; elidrissyhayat@gmail.com (H.E.); yalamiidr@gmail.com (Y.A.I.);; 3 Department of Radiology, University of Pittsburgh, Pittsburgh, PA 15232, USA; 4 Department of Neurology, University of Pittsburgh Medical Center, Pittsburgh, PA 15232, USA; 5 Department of Hematology, University of Pittsburgh Medical Center, Pittsburgh, PA 15232, USA; 6 Department of Neurological Surgery, University of Pittsburgh, Pittsburgh, PA 15232, USA

**Keywords:** insula, olfaction, stimulation, anatomy, neurophysiology, cognitive processing

## Abstract

The insular cortex, a critical hub in the brain’s sensory, cognitive, and emotional networks, remains an intriguing subject of study. In this article, we discuss its intricate functional neuroanatomy, emphasizing its pivotal role in processing olfactory information. Through concise exploration, we delve into the insula’s diverse connectivity and its involvement in sensory integration, particularly in olfaction. Stimulation studies in humans reveal compelling insights into the insula’s contribution to the perception of smell, hinting at its broader implications for cognitive processing. Additionally, we explore an avenue of research in which studying olfactory processing via insular stimulation could unravel higher-level cognitive processes. This innovative approach could help give a fresh perspective on the interplay between sensory and cognitive domains, offering valuable insights into the neural mechanisms underlying cognition and emotion. In conclusion, future research efforts should emphasize a multidisciplinary approach, combining advanced imaging and surgical techniques to explore the intricate functions of the human insula. Moreover, awake craniotomies could offer a unique opportunity for real-time observation, shedding light on its neural circuitry and contributions to higher-order brain functions. Furthermore, olfaction’s direct cortical projection enables precise exploration of insular function, promising insights into cognitive and emotional processes. This multifaceted approach will deepen our understanding of the insular cortex and its significance in human cognition and emotion.

## 1. Introduction

**Figure 1:**
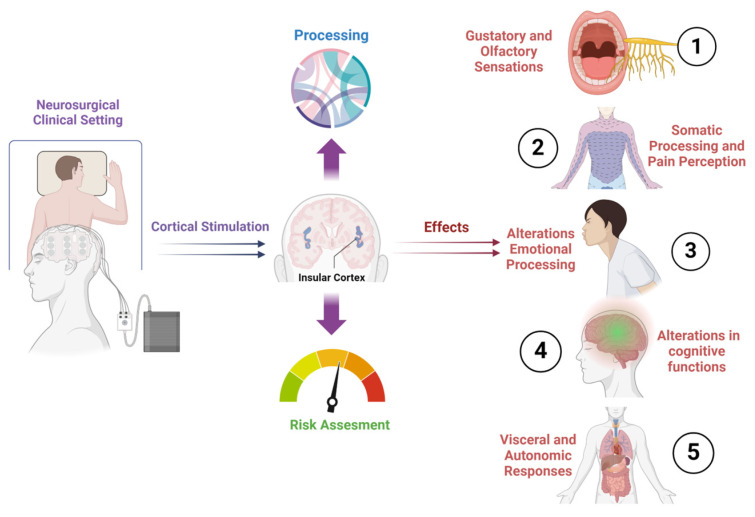
A graphical representation that illustrates the diverse array of effects observed during insular stimulation in humans. Different regions of the insula were selectively stimulated, showcasing distinct outcomes across sensory, emotional, and cognitive domains.

The insular cortex represents a unique, highly interconnected cortical region, where diverse inputs from various brain areas converge. In primates, it is positioned within the fold of the lateral sulcus, while in rodents, it resides on the lateral surface of the brain [1,2,3]. This region can be anatomically divided into posterior and anterior parts, each exhibiting distinct connectivity with other brain areas [4,5]. Cytoarchitecturally, it comprises areas with different characteristics, including a granular area with six layers, an agranular area lacking layer IV, and an intermediate dysgranular area [6]. The anatomical location and diverse architecture of the insula support its multifaceted role as a central hub integrating sensory, cognitive, and emotional processes (Figure 1) [7,8,9].

The insula has been implicated in a broad range of functions from basic sensory processing to higher-order reasoning such as interoceptive awareness and sense of self. For instance, the insula is believed to serve as the primary gustatory cortex [10,11], thermosensory cortex [12], olfactory [11,13], and the visceral sensory cortex [14], as well as integrate sensory perception to yield information about the homeostatic condition of the body. This positions the insula as the primary interoceptive cortex supporting self-monitoring of internal sensation and physiology [2,15,16]. As a representation of the body’s state, the insula is believed to underpin the neurobiological substrate for internal conscious experience, encompassing functions such as self-recognition, emotional feeling states [17,18,19], and meta-cognition, as evidenced by insular cortex activation when viewing images of oneself in sentient species [20] or engaging in aspects of self-reflection and monitoring [21]. This is further supported by its activation during body control awareness, heartbeat awareness, emotional control, and the strong interconnection between the insular cortex and the limbic system [22,23]. In addition to its role in interoceptive attention, insula, specifically the anterior insular cortex (AIC) is also crucial for cognitive control and acts as a bottleneck within the cognitive control network, which is composed of frontoparietal network and cingulo-opercular network including the anterior cingulate cortex (ACC) and AIC [24] Evidence indicates that lesions in the AIC, but not the ACC, significantly impair cognitive control capacity, highlighting the unique role of AIC [24]. The AIC serves as a network hub, facilitating communication and integration across various brain regions and networks.

The insula also plays a significant role in immune regulation, evidenced by early findings that insular damage disrupts acquisition of conditioned immunosuppression in rats [25]. Increased insular activity and altered connectivity are observed in response to pro-inflammatory conditions and diseases such as rheumatoid arthritis and inflammatory bowel disease [26]. Recent studies demonstrate that during colitis and peritonitis, the insular cortex shows elevated neuronal activity, enhancing immune responses in the colon and peritoneum [27]. This immune effect is not primarily pain-mediated, as indicated by partial attenuation with acetaminophen. Inhibition of the insula impairs immune responses and associated inflammatory clinical parameters [26]. Insular activation correlates with systemic inflammatory response manifestations like malaise, pain, and anxiety, and is connected to various brain regions involved in immune regulation [27]. Both right and left anterior insula have roles in immune response, with mixed findings on laterality and subregional activation [26]. 

Of the many known involvements of insula is olfaction which is the specific focus of this article. While the involvement of the insula in olfaction is widely acknowledged, its precise function in olfactory processing remains equivocal. Olfaction is considered a primitive sense due to its evolutionary conservation and direct projections to higher cortical regions, distinguishing it from more elaborate sensory systems. Studies employing insular stimulation have demonstrated alterations in olfactory processing, revealing the pivotal role of the insula in modulating olfactory perception and emphasizing its evolutionary significance as a neural substrate for primitive sensory functions. Olfaction provides a unique avenue for studying the integration of basic sensory experiences by the insula into higher-level conscious thought. For instance, the olfactory perception of coffee not only activates olfactory centers but also triggers mental imagery, including visual, tactile, and gustatory elements. This intricate multisensory integration, believed to be supported in part by the insula, can be probed through olfactory stimuli, enabling a detailed investigation into how the insula processes and amalgamates diverse sensory inputs to construct complex conscious thoughts and mental representations. Furthermore, olfaction stands apart from other sensory modalities as it possesses direct connections to the cortex, circumventing thalamic relay. This unique neural pathway makes olfaction an advantageous tool for studying how information is integrated into the insula. By leveraging olfactory stimuli during research, it becomes feasible to discern the direct cortical processing of olfactory information by the insula, providing insights into the mechanisms involved in the integration of sensory inputs within this cerebral region. In this article, we will review the relationship between the insula and olfaction and the implications of human insula stimulation. 

## 2. Overview of the Functional Neuroanatomy of the Insula

### 2.1. Location of the Insular Cortex

In primates, including humans, the insula is situated in the depths of the lateral sulcus within each hemisphere, concealed beneath portions of the frontal, parietal, and temporal lobes, forming what is known as opercula or ‘lids’ [28]. This distinctive location has given rise to names such as ‘insula’ (derived from the Latin word for ‘island’), ‘hidden fifth lobe’, and ‘Island of Reil’. Macroscopically, the human insula is partitioned into anterior and posterior segments by the central insular sulcus [6]. The endpoints of these segments exhibit notable differences in connectivity to other brain regions, while an intermediate ‘middle’ insular zone displays a combination of anterior and posterior connectivity features. In species with smooth brains, such as mice and rats, the insula is exposed on the lateral surface of the hemisphere, primarily above the rhinal fissure.

### 2.2. Insular Cytoarchitecture

The insula, a component of the isocortex (neocortex), exhibits a layered structure comprising six distinct layers. Brodmann’s classification delineates the insula into two regions, distinguished by the central sulcus: an anterior agranular region housing pyramidal neurons in layers II and IV, and a posterior granular region containing granular cells in layers II and IV [6,28]. Subsequent studies introduced a concentric model, situating the agranular region ventral-anteriorly and the granular region dorsal-posteriorly, with a dysgranular cortex band in between [29,30]. While some variability exists, contemporary research generally supports the concentric model. Ongoing investigations into structural parcellation revealed seven subdivisions in the human insula and 15 in the macaque monkey [31].

An intriguing feature of the insula is the presence of von Economo neurons (VENs), large bipolar projection neurons, predominantly found in the frontoinsular cortex [32]. Distinguished by their size and unique morphology, VENs are proposed to function as a rapid relay transmission system, projecting to various brain areas. Postmortem studies in humans indicate that VENs express transcription factors associated with interoceptive functions and deep brain structures [33,34]. The co-expression of additional transcription factors suggests potential secondary projections to intracortical regions.

### 2.3. Connectivity of the Insular Cortex

The insular cortex stands as a pivotal anatomical integration hub, exhibiting robust connectivity with an expansive network of cortical and subcortical brain regions, each contributing to diverse sensory, emotional, motivational, and cognitive functions [1,2,3,15]. Sensory inputs from all modalities are prominently received, facilitated by direct thalamic and horizontal cortical afferents conveying external information (auditory, somatosensory, olfactory, gustatory, and visual) and internal signals (interoceptive information) [2,7,15,35]. These inputs project to organized insular sensory regions, delineating specialized domains such as the ‘visceral insular cortex’, the ‘gustatory cortex’ (primary taste cortex), and insular auditory and somatosensory fields [8,16,20]. Notably, each sensory region within the insula exhibits substantial connectivity with other areas, emphasizing its integrative nature [36,37].

In addition to sensory afferents, the insula engages in reciprocal connections with the limbic system [38,39]. The lateral and basolateral amygdala notably project to granular and dysgranular insular regions, establishing dense efferents to amygdala nuclei [40,41,42]. Further connections extend to the lateral part of the bed nucleus of the stria terminalis, mediodorsal thalamus, lateral hypothalamus, and parahippocampal regions, encompassing the perirhinal and lateral entorhinal cortices [43]. The connectivity between the insula and subcortical structures involved in the regulation of emotion supports its role in the processing of emotional experience and potentially integrating it with other sensory and physiological functions. Recent research highlights the insula’s involvement in assigning the valence of emotional experiences such as aversive emotions, including fear and anxiety [17,18,19], as well as positive emotions, such as happiness. The experience of positive versus negative emotion appears anatomically organized in the insula with the left hemisphere associated with positive emotion and the right hemisphere with negative emotion [17,18,19].

Reciprocal connectivity also extends to frontal brain regions, including the anterior cingulate, orbitofrontal, and medial prefrontal cortices, associated with cognitive, emotional, and executive functions [28,44,45]. Moreover, the insula projects to brain regions linked to motivation and reward, such as the nucleus accumbens and caudate putamen [1,15,28,39,46,47]. Notably, the insular cortex receives substantial neuromodulatory input from cholinergic, dopaminergic, serotonergic, and noradrenergic afferents, contributing to its dynamic and integrative functions [4,6,28]. For instance, Nench et al. [48] induced an ecstatic aura via electrical stimulation of the dorsal anterior insula during presurgical invasive intracerebral monitoring in a 51-year-old patient with epilepsy. This case augments the existing evidence, substantiating the anterior insula’s pivotal role as the principal generator of mystical-type experiences. Furthermore, the human Insular cortex is implicated in higher executive functioning, which is a neurocognitive phenomenon crucial for integrating sensory, emotional, and cognitive information, thereby facilitating higher-level awareness and decision-making processes [20]. A specialized type of neurons named Von Economo neurons, identified within the insular cortex, are specialized projection neurons distinguished by their large size and unique morphology [49]. These neurons have been implicated in orchestrating complex cognitive functions and are hypothesized to play a distinctive role in supporting higher-level cognition, particularly in the realms of social intelligence, emotional processing, and self-awareness. The presence and characteristics of von Economo neurons underscore their potential significance in explaining the neurobiological basis of advanced cognitive capacities and executive functions within the human brain.

## 3. The Role of the Insular Cortex in Sensory Processing

The insular cortex plays a pivotal role in sensory processing, integrating information from diverse afferents to contribute to the overall neural representation of an individual’s internal and external environment [3,8,16]. As a complex neural hub, the insula has been shown to be involved in processing gustatory, olfactory, somatosensory, auditory, and vestibular stimuli in cortical stimulation studies using current-regulated neurostimulators, demonstrating its multimodal sensory functions [2,47,50].

### 3.1. Somatic Processing and Pain

Electrical stimulation of the insular cortex predominantly elicits somatosensory manifestations, including paresthesia and painful sensations [51]. Non-painful tactile and painful stimulation in neuroimaging studies consistently activates the insula [52]. The posterior insula is particularly implicated in thermosensory function and pain perception, responding to noxious stimuli across modalities and body parts [53,54]. Strokes or resections affecting the posterior insula and innermost parietal operculum have been linked to central pain syndromes [55,56].

### 3.2. Visceral Sensations, Autonomic Control, and Interoception

Early reports highlighted a significant number of visceral responses triggered by direct electro-cortical stimulation of the insula [14,57]. Recent tract-tracing studies have further supported the notion of the insula serving a central role in viscero-somatosensory processing [15,58,59]. It receives visceral afferent projections conveying interoceptive information from various bodily regions. Confirming earlier findings, subsequent studies demonstrated unpleasant visceral sensations, including discomfort and painful paresthesia, along with motor responses like nausea and vomiting [11,60]. This visceral role suggests potential involvement in autonomic function regulation, supported by observed heart rate and blood pressure changes following insular stimulation and lesions.

Beyond visceral processing, the insula’s role in interoception, the sense of the body’s physiological condition, has been proposed. Functional neuroimaging studies revealed heightened insular activation when individuals became aware of thirst, heartbeat, and organ distention [61,62]. Lesions in the insula were associated with delayed awareness of cardiovascular sensations and anosognosia for hemiplegia/hemiparesis. The insula’s posterior-to-anterior progression in integrating visceral information supports the creation of refined perceptual maps of the organism’s state.

### 3.3. Auditory Processing

Given its efferent projections from primary auditory areas, the insular cortex shows involvement in central auditory processing [63,64]. Electrical stimulation produces auditory responses, mainly illusions, and distortions, in the lower posterior insula [65]. Functional neuroimaging studies and cases with isolated insular lesions indicate central auditory deficits, including temporal resolution and sequencing deficits, highlighting the insula’s role in auditory intensity processing [66].

### 3.4. Chemosensory Functions

The insula is involved in processing gustatory stimuli, with the primary gustatory area located in the anterior insula and adjoining frontal operculum. Electrical stimulation of specific insular regions may evoke gustatory hallucinations [67]. Studies have reported taste deficits following insular damage [68]. The insula also plays a role in olfaction, consistently activated in response to olfactory stimuli. Case studies suggest its involvement in modulating the intensity of olfactory stimuli [11,69].

Olfactory Function: Structural neuroimaging investigations indicate that cortical characteristics of the bilateral insulae correlate with olfactory sensitivity [70]. Enhanced thickness and density of the insular cortex in healthy individuals are linked to superior olfactory performance, while patients experiencing various degrees and forms of olfactory dysfunction exhibit gray matter reduction in the insula [71]. Functional neuroimaging studies reveal consistent insular activation in response to diverse olfactory stimuli, with hemispheric differences implicated in valence processing [11]. The right hemisphere appears specialized for pleasant odors, while the left hemisphere may exhibit heightened responsiveness to unpleasant odors across modalities [72,73,74]. Electro-cortical stimulation studies of the insular cortex align with these patterns, reporting a tendency for insula stimulation to evoke unpleasant olfactory sensations [11]. Additionally, a functional neuroimaging study indicated that the magnitude of the insular response may differ based on the quality of food odors: the sweeter the perceived scent of a food odor, stronger the insular reaction [75].

### 3.5. Olfaction as a Fundamental and Primitive Sense

Olfaction constitutes an ancient and evolutionarily crucial physiological system. In humans, chemosensation plays a pivotal role in safety, nutrition, pleasure perception, and overall well-being. Olfactory information generated is processed and encoded in the olfactory bulb, disseminating to various brain areas. The discovery of olfactory receptors has significantly enhanced our molecular understanding; however, the intricate processes involved in translating varied odorant molecules and the capacity of the olfactory system to discern a broad range of smells into coherent signals for the brain continue to be subject to rigorous investigation [76]. Challenges arise from the intricate neural networks involved in olfactory sensation. Consequently, our understanding of olfactory dysfunction in humans is rudimentary but poses a potential opportunity for studying the neural circuity of integrating sensory functions in the insula. 

Within the olfactory epithelium, an estimated 10 to 20 million olfactory neurons are interspersed amid various supportive cells [77]. This pseudostratified columnar epithelium comprises basal cells, recognized (in animals, with inconclusive evidence in humans) as potential stem cells capable of generating all epithelial components [78]. Additionally, it encompasses Bowman’s glands, microvillar cells, and sustentacular cells, hypothesized to support olfactory neuron function. Bowman’s acini, characterized as exocrine, produces vital substances for olfaction. Central to olfactory mucus are odorant-binding proteins, acting as chaperones to facilitate odorant–receptor interactions [77]. While Bowman’s glands are well-understood contributors, the precise roles of other cell types in supporting neuronal function, likely through less-defined mechanisms, necessitate further elucidation, potentially involving the provision of an optimal local environment for signal transduction [77].

### 3.6. Olfactory Responses to Stimulation of the Human Insula

Stimulation studies of the insula involving human subjects have documented the involvement of the insula in the perception of smell. For instance, Mazzola et al. [11] analyzed 651 recordings of electrical stimulation applied to the insula in 221 patients (107 females and 114 males with a mean age of 35.5 years), utilizing stereotactically implanted depth electrodes as part of the presurgical evaluation for drug-refractory epilepsy. They found that 550 patients (84%) produced a clinical response in the form of undefinable taste in the mouth, bad mouth taste, acid taste, salty taste when stimulating the left insula and bad taste on the right side of the tongue, metallic taste, metallic taste associated with mouth paranesthesia and unidentifiable taste with jaw paranesthesia when stimulating the right insula, 237 (43%) from right stimulation, and 313 (57%) from left insular stimulation. Fifteen gustatory responses (2.7%) and 6 olfactory responses (1.1%) were evoked. In addition, numerous neuroimaging investigations in humans, employing functional magnetic resonance imaging (fMRI) or positron-emission tomography (PET), have consistently documented activations within various sectors of the insular cortex in response to gustatory and olfactory stimulations [79,80,81]. The involvement of the insula in the processing of gustatory sensations (GSs) and olfactory sensations (OSs) is substantiated by a wealth of data, encompassing anatomical investigations in nonhuman primates and cerebral lesion studies in humans [82,83,84]. 

In the simulation study by Mazzola et al. [11], the team also showed that there is a convergence of gustatory and olfactory regions within the mid-dorsal insula. This observation potentially elucidates challenges faced by certain patients in distinguishing between gustatory and olfactory sensations, leading to occasional confusion at the conscious level. Notably, instances were noted where a reported “metallic sensation” was interpreted by patients as either a taste or a smell highlighting the interconnectedness of regions within the insula. While traditional human taste sensations include sweet, salty, sour, and bitter, qualities such as a “metallic sensation” documented in insular stimulation studies may be related to the diverse receptors for olfaction [85,86]. 

### 3.7. Can Studying Olfactory Processing via Stimulation of the Human Insula Inform Higher-Level Cognitive Processes

Exploring olfactory processing through stimulation of the human insula could hold the potential for informing higher-level cognitive processes. The insula, a multifaceted brain region, is implicated not only in olfactory perception but also in various cognitive and emotional functions. By investigating the effects of insular stimulation on olfactory processing, researchers may uncover insights into the intricate interplay between sensory and cognitive domains. This approach could offer a unique perspective on how olfactory information is integrated into broader cognitive frameworks, shedding light on the neural mechanisms that contribute to higher-level cognitive processes. The endeavor to understand the functional role of the insula in olfactory cognition may unveil connections that extend beyond sensory perception, providing valuable contributions to the broader field of cognitive neuroscience. Moreover, olfaction as a function presents a unique advantage in scientific study compared to other sensory modalities due to its distinctive neural pathway. Unlike other sensory functions such as vision and audition, olfactory signals exhibit a direct connection to the cortex, bypassing the thalamus [87,88]. The olfactory nerve projects directly from the nasal epithelium to the olfactory bulb, where initial processing occurs, and subsequently, information is conveyed directly to higher-order cortical regions, particularly the olfactory cortex. This direct connection allows for a more straightforward and rapid transmission of olfactory information, facilitating a more accessible investigation of olfactory processing mechanisms compared to sensory modalities that necessitate thalamic relay [89]. The direct cortical projection in olfaction streamlines the study of odor perception and neural processing, providing researchers with a clearer and more direct route to explore the intricacies of olfactory sensation.

## 4. Conclusions and Future Directions

In summary, the insula is implicated in a myriad of functions, with studies across species revealing its complex yet central role in connecting various sensory, emotional, motivational and cognitive processing. Its connectivity makes it adept at monitoring the current environment, and emotional and bodily states, and predicting how future actions impact survival and well-being based on experience. This centrality is crucial for determining the valence of internal and external stimuli, and explaining its roles in reinforcement learning, emotion control, and decision-making. Additionally, the insula is proposed as a salience detector, highlighting the most relevant stimuli for further processing in other brain networks. Beyond these general roles, the insula comprises distinct subregions with different connectivity patterns and seemingly diverse functions, raising questions about their interaction and whether common neuronal computations underlie seemingly distinct functions or if they operate as separate modules, crucial for advancing our understanding of insula function.

The future direction of research on the insular cortex should involve a multidisciplinary approach aimed at gaining a more comprehensive understanding of its intricate functions and contributions to various physiological and psychological processes combining advanced imaging technologies, and connectomics to further explore the intricate connectivity patterns of the insular cortex with other brain regions. The study of the insula during awake brain surgery could offer a unique opportunity to investigate its functional roles in cognition and emotion. Real-time observation of this cortical region could provide insights into its intricate neural circuitry, contributing to a comprehensive understanding of higher-order brain functions in vivo. The current limited exploration of the insula stems from the challenging nature of the neurosurgical procedure, demanding a high level of expertise. Its deep-seated location and intricate connections necessitate specialized skills, making studies during surgery uncommon. Advancements in surgical techniques and expertise may now facilitate more comprehensive investigations. Additionally, the direct olfactory pathway to the cortex, bypassing the thalamus, enables studying insular function during awake brain surgeries. This pathway offers a direct way to investigate olfactory processing, providing a tangible method to explore the role of insular cortex in cognition and emotion in real time. This approach contributes to understanding the complex functions of the insular cortex in human cognition, emotion, and health.

## Data Availability

Not applicable.
